# An enriched environment improves maternal sleep deprivation‐induced cognitive deficits and synaptic plasticity via hippocampal histone acetylation

**DOI:** 10.1002/brb3.3018

**Published:** 2023-04-18

**Authors:** Yue‐Ming Zhang, Ru‐Meng Wei, Ming‐Zhu Ni, Qi‐Tao Wu, Yun Li, Yi‐Jun Ge, Xiao‐Yi Kong, Xue‐Yan Li, Gui‐Hai Chen

**Affiliations:** ^1^ Department of Neurology (Sleep Disorders) the Affiliated Chaohu Hospital of Anhui Medical University Hefei Anhui P. R. China

**Keywords:** enriched environment, histone acetylation, learning and memory, maternal sleep deprivation

## Abstract

**Introduction:**

Growing evidence clearly demonstrates that maternal rodents exposure to sleep deprivation (SD) during late pregnancy impairs learning and memory in their offspring. Epigenetic mechanisms, particularly histone acetylation, are known to be involved in synaptic plasticity, learning, and memory. We hypothesize that the cognitive decline induced by SD during late pregnancy is associated with histone acetylation dysfunction, and this effect could be reversed by an enriched environment (EE).

**Methods:**

In the present study, pregnant CD‐1 mice were exposed to SD during the third trimester of pregnancy. After weaning, all offspring were randomly assigned to two subgroups in either a standard environment or an EE. When offspring were 3 months old, the Morris water maze was used to evaluate hippocampal‐dependent learning and memory ability. Molecular biological techniques, including western blot and real‐time fluorescence quantitative polymerase chain reaction, were used to examine the histone acetylation pathway and synaptic plasticity markers in the hippocampus of offspring.

**Results:**

The results showed that the following were all reversed by EE treatment: maternal SD (MSD)‐induced cognitive deficits including spatial learning and memory; histone acetylation dysfunction including increased histone deacetylase 2 (HDAC2) and decreased histone acetyltransferase (CBP), and the acetylation levels of H3K9 and H4K12; synaptic plasticity dysfunction including decreased brain‐derived neurotrophic factor; and postsynaptic density protein‐95.

**Conclusions:**

Our findings suggested that MSD could damage learning ability and memory in offspring via the histone acetylation pathway. This effect could be reversed by EE treatment.

## Graphical abstract

The study showed that maternal sleep deprivation could damage learning ability and memory in offspring via the histone acetylation pathway. This effect could be reversed by enriched environment treatment

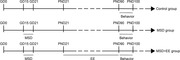

## 1 INTRODUCTION

Sleep is a basic activity of life, and long‐term sleep insufficiency can lead to psychological problems ranging from mood changes to psychotic symptoms and cognitive impairment (Krause et al., 2017). Probably owing to anatomic, physiological, and hormonal changes, about half of pregnant women complain about their sleep pattern, which is characterized by hyperarousal, sleep fragmentation, poor sleep quality, and insomnia; this increases gestational week, and is especially prevalent in the third trimester (Mindell et al., 2015; Sedov et al., 2018; Wilson et al., 2011). Previous studies suggested that sleep loss during pregnancy not only increases the risk of psychological issues in mothers, but also disrupts the intrauterine environment and affects the development of the fetal nervous system, leading to offspring frequently exhibiting cognitive impairment in adulthood (Peng et al., 2016; Wu et al., 2014). Due to ethical limitations, sleep deprivation (SD) could not be performed in normal pregnant women to study the behavioral phenotypes in offspring. So, it is necessary to use a rodent sleep restriction protocol to simulate the abnormal sleep patterns of pregnant women to explore the molecular and biological mechanisms underlying cognitive deficits in offspring.

The hippocampus is a pivotal area well‐known for its role in learning and memory processes and is vulnerable to stress. Mounting evidence suggests that decreased hippocampal neurogenesis and microglial activation are associated with cognitive impairment in offspring caused by maternal SD (MSD) (Zhao et al., 2014, 2015). Hippocampal synaptic plasticity with long‐term potentiation (LTP) reflects persistent changes in synaptic strength and is the cellular basis of learning and memory (Duchon et al., 2020; Martín‐Rodríguez et al., 2020; Matynia et al., 2002). Several findings show that MSD results in impaired hippocampal LTP at Schaffer collaterals/CA1 synapses and decreased basal synaptic transmission in the hippocampal CA1 region of the offspring (Peng et al., 2016). The formation of LTP requires the activation of calmodulin‐dependent protein kinases and the translocation of α‐amino‐3‐hydroxy‐5‐methyl‐4‐isoxazole‐propionic acid (AMPA) receptors to postsynaptic membranes, which are regulated by synaptic plasticity‐associated proteins (Deak & Sonntag, 2012; Foster, 2012). Brain‐derived neurotrophic factor (BDNF) and postsynaptic density protein‐95 (PSD‐95) are synaptic plasticity‐associated proteins. BDNF, which is mainly expressed in the hippocampus, can promote calcium influx and AMPA receptor transfer to postsynaptic membranes (Chakravarthy et al., 2006; Wu et al., 2016), while PSD‐95 is a highly stable synapse protein that binds to *N*‐methyl‐d‐aspartic acid receptor (NMDA) receptors and stabilizes them in postsynaptic neurons (Lin et al., 2004). However, whether MSD‐induced impaired LTP involves alterations in BDNF and PSD‐95 and the underlying mechanisms remain unclear.

Enriched environment (EE), a protocol that exposes rodents to motor, sensory, and social interaction stimulation by placing them in a larger environment containing different toys, is an effective way to improve cognitive impairment (Keymoradzadeh et al., 2020; Mohammadian et al., 2019; Yu et al., 2020). It is widely accepted that EE can increase the expression of BDNF and nerve growth factor to regulate cell survival, synaptogenesis, neurogenesis, and dendritic arborization (Alwis & Rajan, 2014). Importantly, accumulating evidence confirms that EE improves synaptic activity, signaling, LTP, and spatial learning and memory (Wang et al., 2020; Zarif et al., 2018; Zhang et al., 2021). The beneficial effects of EE have been identified in various animal models of disorders including Parkinson's disease, Alzheimer's disease, traumatic brain injury, stroke, and Huntington's disease (Cattaud et al., 2018; Couly et al., 2021; Schreiber et al., 2014; Wi et al., 2018; Yuan et al., 2021).

Epigenetics is a bridge linking behavioral phenotypes to environmental stimulations by altering gene transcription without altering gene sequence (Kim & Kaang, 2017). Epigenetic modifications, especially histone acetylation, have been implicated in synaptic plasticity, learning, and memory (Schueller et al., 2020). The balance of histone acetylation is controlled by histone acetyltransferase and histone deacetylase. Histone acetyltransferases, including CBP (CREB‐binding protein) and P300, add acetyl groups to lysine sites of chromatin that recruit transcription factors that, in turn, can activate gene transcription. Inversely, histone deacetylases, especially HDAC2 (histone deacetylase 2), repress gene transcription through removing acetyl groups and tightening the chromatin structure (Mahgoub & Monteggia, 2014; Sen, 2015). Dysfunction of histone acetylation has been observed in various pathological models of cognitive impairment, which could be improved by EE or HDAC inhibitors (Gräff & Tsai, 2013; Neidl et al., 2016; Wang et al., 2016).

In this study, we hypothesized that EE could improve MSD‐induced cognitive impairment through modulating histone acetylation. To prove it, the MSD model was created in which mice received SD by activity wheel during the third trimester and randomly assigned to a standard environment or an EE. The adverse effect of MSD on the cognitive function of offspring mice was evaluated. In addition, the levels of histone acetylation markers and synaptic plasticity‐associated proteins, such as BDNF and PSD‐95, were measured in the hippocampus.

## 2 MATERIALS AND METHODS

### 2.1 Animals

**FIGURE 1:**
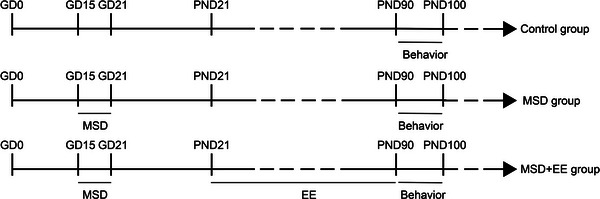
Experimental protocol. GD, gestational day; PND, postnatal day; MSD, maternal sleep deprivation; EE, enriched environment.

CD‐1 mice (2‐month‐old, Vital River Laboratory Animal Technology Co., Ltd., Beijing, P.R. China) adapt to the environment by being housed for 2 weeks. The male and female CD‐1 mice mated 1:2 at 21:00 h in a cage, and the presence of a vaginal plug the following morning was taken as day 0 of gestation (GD 0). Pregnant CD‐1 mice were housed in individual cages maintained in a temperature‐controlled room (22−25°C), and kept on a 12‐h light/dark cycle with water and food available ad libitum. Pregnant mice underwent SD during late pregnancy (GD 15−21) or had no intervention. The offspring of these pregnant mice were used as this study's subjects. All offspring were randomly divided into three groups after being separated from their dams on the 21st day after birth, control group (Control group), maternal SD group (MSD group), and maternal SD plus enriched environment group (MSD + EE group). At 3‐month‐old, eight male or female mice in each group were used for behavioral, fluorescence real‐time quantitative PCR, and western blot analyses (see Figure 1). All animal experiments were performed following the protocols approved by the Laboratory Animal Committee of Anhui Medical University.

### 2.2 SD procedure

Given that sleep insufficiency occurs mainly in the third trimester during pregnancy and 6 h of SD in the daytime mimic insomnia symptoms well, we created the MSD model with 6 h of SD per day in the third trimester according to the previous literatures (Peng et al., 2016; Yu et al., 2018). SD was performed using a special apparatus (BW‐NSD404, Shanghai Bio‐will Co., Ltd., P. R. China) for 6 h (12:00–18:00 h) per day for the MSD + EE and MSD groups. The SD apparatus ensured that mice remained awake through a constantly moving activity wheel, set at 0.5 m/min. Water and food were supply available during throughout the SD period.

### 2.3 Enriched environment

From day 21 to 3 months of age, the MSD + EE group mice were placed in larger cages (52 × 40 × 20 cm) with eight mice per cage; cages contained a variety of toys which changed every 2 weeks such as platforms, a wood shelter, running wheels, stepladders, and plastic tunnels. The objects were changed twice a week to maintain environmental novelty. Mice from the Control group and MSD group were raised in standard cages (36 × 18 × 14 cm) with three mice per cage without objects.

### 2.4 Morris water maze test

The Morris water maze test was used to assess spatial learning and memory abilities in the 3‐month‐old offspring mice (eight mice per group). Briefly, a black round water tank (diameter 150 cm, height 30 cm) was filled with 20–21°C water and surrounded by a white curtain with three different patterns (triangular, round, square). A black fixed platform (10 cm in diameter and 24 cm in height) 1.0 cm below the water surface. Each mouse was tested four times a day for 7 days. The order of water entry points is different every day, but the position of the platform is fixed. Mice were allowed to find the hidden platform within 60 s. After finding the platform, the mice stayed for 30 s before the next experiment. If the mice fail to find the platform within 60 s, they will be guided to this platform. Learning ability was assessed by the latency (the time it takes to find the target platform), while memory ability was assessed by the percentage of time it takes in the target quadrant. All data were recorded using ANY‐maze software (Stoeling, USA).

### 2.5 Western blotting

Western blotting uses the right hippocampal tissue. The tissue protein was extracted by adding 600 μL of RIPA cell lysate (containing 0.6 mM PMSF) to tissue samples for lysis. Centrifugation took place at 12,000 rpm for 15 min and the supernatant was collected. Subsequently, 5x SDS–PAGE protein loading buffer (1:4) was added to the collected protein samples, and protein is denaturated in boiling water bath for 15 min. Then, the protein samples were loaded into the loading well of the SDS–PAGE gel with 5–10 μL per well, and electrophoresis took place at a constant pressure of 80 v for approximately 1 h. Then, constant flow transfer membranes were used with transfer times of 50, 60, 40, 20, 20, and 20 min for HDAC2, PSD‐95, CBP, mature BDNF, acetyl‐H3k9, and acetyl‐H4K12, respectively. Subsequently, the protein membranes were rinsed, blocked, and incubated with rabbit antibodies of anti‐HDAC2 (1:2,000; Abcam, ab32117), anti‐PSD‐95 (1:2,000; Abcam, ab238135), anti‐CBP (1:1,000; Bioworld, BS1624), anti‐mature BDNF (1:1,000; Abcam, ab108319), anti‐acetyl‐H3k9 (1:5,000; Abcam, ab177177), and anti‐acetyl‐H4K12 (1:10,000; Abcam, ab177793) overnight at 4°C. Secondary antibodies were incubated with horseradish peroxidase‐labeled goat anti‐rabbit IgG (1:20000; Zsbio, ZB‐2301). The ImageJ software (Media Cybernetics, USA) was used to analyze the protein bands.

### 2.6 Real‐time fluorescence quantitative PCR

The left hippocampus was used for RNA extraction following the experimental instructions. RNA was used for cDNA synthesis according to a RT‐PCR kit (TaKaRa, RR047A). cDNA was used as a template for fluorescence quantification. The RT‐PCR procedure was performed at 95°C for 60 s, followed by 40 cycles at 95°C for 20 s, and 60°C for 60 s. Analysis took place using relative quantification, and the calculation method was 2^−△△^ *^Ct^*. The primer sequences are shown in Table 1.

**TABLE 1:** Primer sequences.

Gene	Forward primer (5′→3′)	Reverse primer (5′→3′)
β‐actin	AGTGTGACGTTGACATCCGT	TGCTAGGAGCCAGAGCAGTA
CBP	GTGAAGATGGCCGAGAACTT	CAGCTCATCAGGAAGGTCAT
BDNF	TTACTCTCCTGGGTTCCTGA	ACGTCCACTTCTGTTTCCTT
PSD‐95	CCCAGGATATGTGAACGGAA	CCTGAGTTACCCCTTTCCAA
HDAC2	TATCCCGCTCTGTGCCCTA	CCTCCTTGACTGTACGCC

### 2.7 Statistical analyses

The results of this study were calculated by Graphpad Prism 8.0. All results were presented as the mean ± standard error of the mean. The statistical analyses of the Morris water maze test results is adopted repeated‐measure ANOVA followed by Tukey's *post‐hoc* test. Differences in group and sex of biochemical outcomes were analyzed using a two‐way ANOVA, followed by Tukey's *post‐hoc* test. *p* < .05 was accepted as statistically significant.

## 3 RESULTS

### 3.1 An EE reversed cognitive impairment induced by maternal SD

**FIGURE 2:**
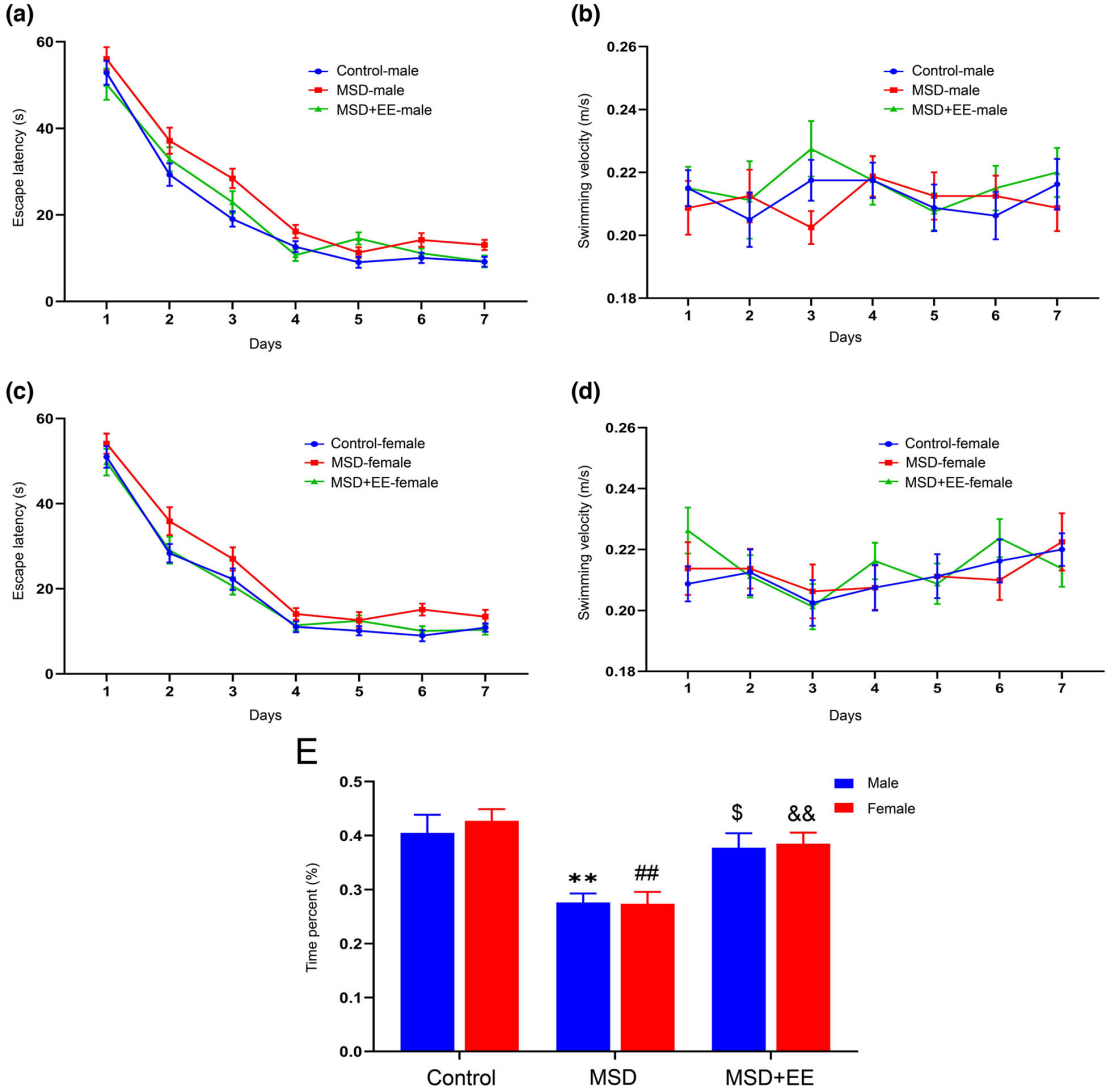
EE reversed the deficits in spatial learning and memory ability induced by MSD. The escape latency **(a, c)** and swimming velocity **(b, d)** in the Morris water maze test are shown. The percentage of time spent in the target quadrant in the probe test is shown **(E)**. ^**^ *p* < .01 versus control male; ^##^ *p* < .01 versus control female; ^$^ *p* < .05 versus MSD male; ^&&^ *p* < .01 versus MSD female.

In the acquisition phase, the escape latency showed both significant effects of time and treatment (time: *F*_(6,252)_ = 270.44, *p* < .001, treatment: *F*_(2,42)_ = 15.39, *p* < .001, sex: *F*_(1,42)_ = 0.60, *p* > .05; Figure 2a–d). However, no such effect was observed with time × treatment (*F*_(12,252)_ = 1.25, *p* > .05), time × sex (*F*_(6,252)_ = 0.40, *p* > .05), time × treatment × sex (*F*_(12,252)_ = 0.36, *p* > .05). Further analysis revealed that MSD‐induced male and female offspring spent more time finding the hidden platform than the Control group and MSD + EE group. No significant swimming velocity changes were observed in any of the three groups.

In the retention phase, the time spent in the target quadrant showed significant differences between the three groups (treatment: *F*_(2,42)_ = 18.60, *p* < .001, sex: *F*_(1,42)_ = 0.22, *p* > .05, treatment × sex: *F*_(2,42)_ = 0.14, *p* > .05, Figure 2e). *Post‐hoc* comparisons revealed that the percent of time spent swimming for both males and females of MSD group was less than in the Control group and MSD + EE group. These results suggest that, EE could improve impaired hippocampal learning and memory induced by MSD.

### 3.2 Effects of maternal SD and EE on histone acetylation, HDAC2, and CBP

**FIGURE 3:**
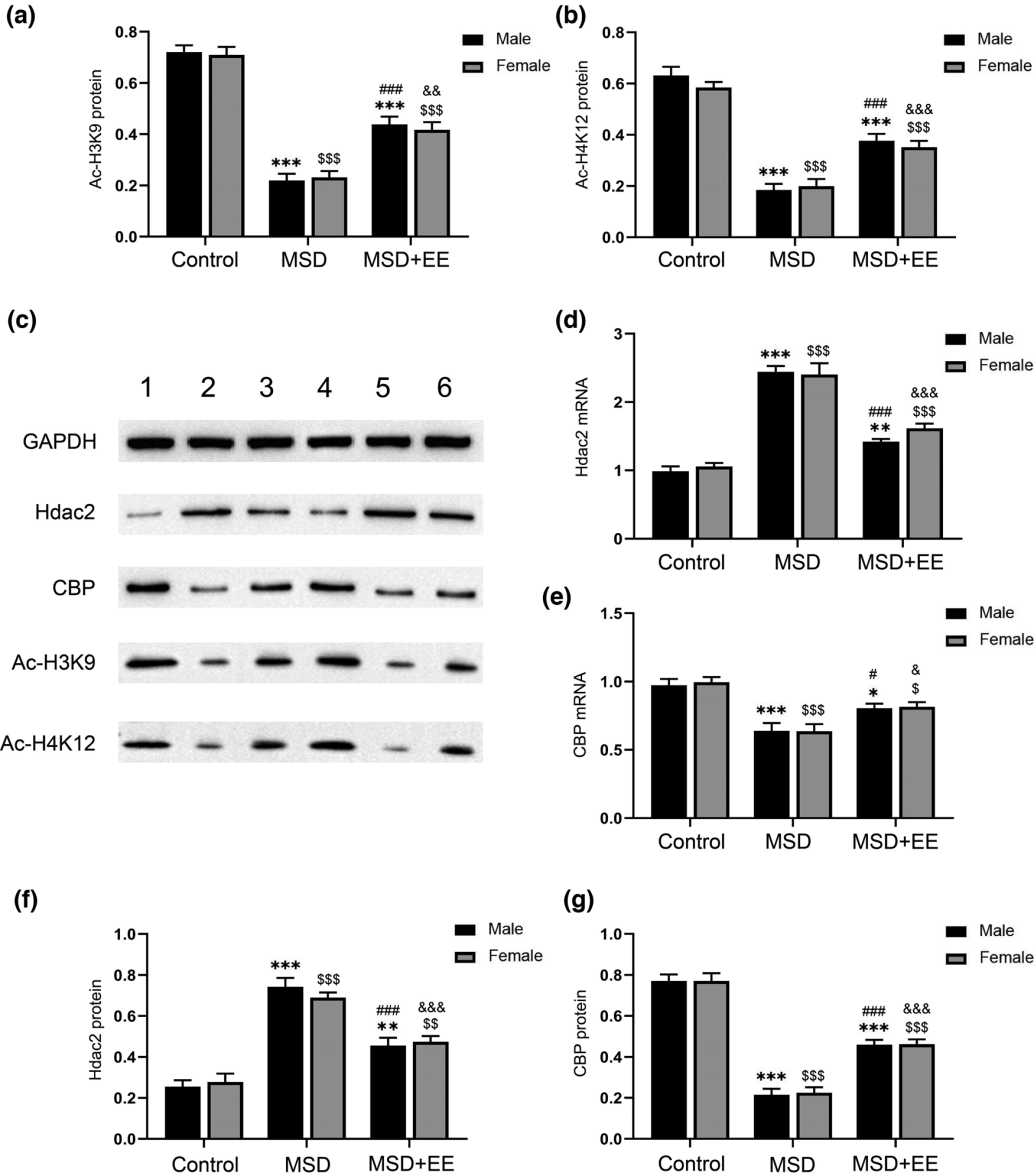
Effects of MSD and EE on the expression levels of histone acetylation indicators. **(a, b)** The protein levels of acetyl‐H3K9 (Ac‐H3K9) and acetyl‐H4K12 (Ac‐H4K12) in the hippocampus. **(c)** Western blot: band 1: Control‐male; band 2: MSD‐male; band 3: MSD + EE‐male; band 4: Control‐female; band 5: MSD‐female; band 6: MSD + EE‐female. **(d, e)** The mRNA levels of HDAC2 and CBP in the hippocampus. **(f, g)** The protein levels of HDAC2, CBP in the hippocampus. ^*^ *p* < .05, ^**^ *p* < .01, ^***^ *p* < .001 versus control male; ^$^ *p* < .05, ^$$^ *p* < .01, ^$$$^ *p* < .001 versus control female; ^#^ *p* < .05, ^###^ *p* < .001 versus MSD male; ^&^ *p* < .05, ^&&^ *p* < .01, ^&&&^ *p* < .001 versus MSD female.

We investigated the effect of EE and MSD on chromatin histone acetylation. The protein levels of acetyl‐H3K9 and acetyl‐H4K12 were significantly different among the three groups (acetyl‐H3K9: treatment: *F*_(2,30)_ = 152.30, *p* < .001, sex: *F*_(1,30)_ = 0.08, *p* > .05, treatment × sex: *F*_(2,30)_ = 0.18, *p* > .05; acetyl‐H4K12: treatment: *F*_(2,30)_ = 123.80, *p* < .001, sex: *F*_(1,30)_ = 0.76, *p* > .05, treatment × sex: *F*_(2,30)_ = 0.69, *p* > .05; Figure 3a–b). The *post‐hoc* analysis revealed that MSD decreased the protein levels of acetyl‐H3K9 and acetyl‐H4K12 in the hippocampus, while EE reversed it. Histone acetylation was catalyzed by HAT and reversed by HDAC. Therefore, we examined the mRNA and protein levels of HDAC2 and CBP. The mRNA and protein levels of HDAC2 and CBP were significantly different among the three groups (mRNA: HDAC2: treatment: *F*_(2,42)_ = 126.50, *p* < .001, sex: *F*_(1,42)_ = 1.05, *p* > .05, treatment × sex: *F*_(2,42)_ = 0.85, *p* > .05; CBP: treatment: *F*_(2,42)_ = 29.60, *p* < .001, sex: *F*_(1,42)_ = 0.07, *p* > .05, treatment × sex: *F*_(2,42)_ = 0.04, *p* > .05; Figure 3d and e; protein: HDAC2: treatment: *F*_(2,30)_ = 82.89, *p* < .001, sex: *F*_(1,30)_ = 0.01, *p* > .05, treatment × sex: *F*_(2,30)_ = 0.85, *p* > .05; CBP: treatment: *F*_(2,30)_ = 183.40, *p* < .001, sex: *F*_(1,30)_ = 0.03, *p* > .05, treatment × sex: *F*_(2,30)_ = 0.02, *p* > .05; Figure 3f and g). The *post‐hoc* analysis revealed that MSD‐induced offspring's cognitive impairment was associated with an increased HDAC2 mRNA and protein and a decreased CBP mRNA and protein, while EE reversed it.

### 3.3 Effects of an enriched environment and maternal SD on BDNF and PSD‐95

**FIGURE 4:**
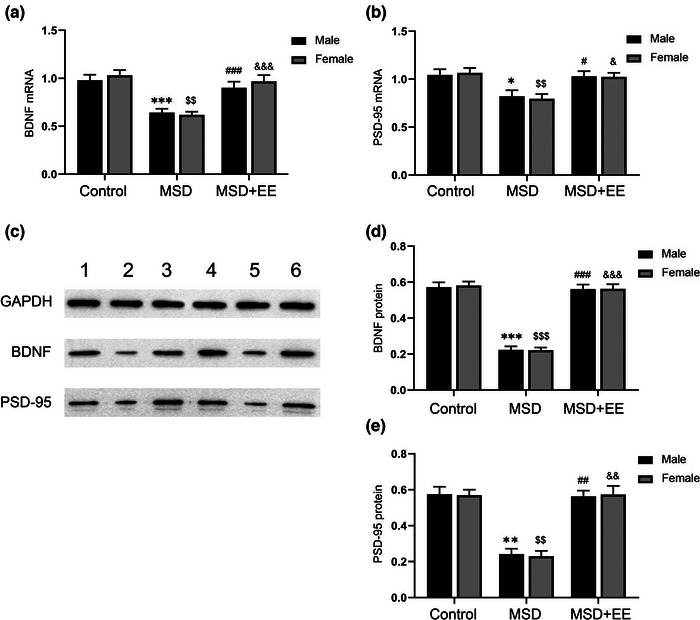
Effects of MSD and EE on the expression levels of synaptic plasticity markers. **(a, b)** The mRNA levels of BDNF and PSD‐95 in the hippocampus. **(c)** Western blot: band 1: Control‐male; band 2: MSD‐male; band 3: MSD + EE‐male; band 4: Control‐female; band 5: MSD‐female; band 6: MSD+EE‐female. **(d, e)** The protein levels of BDNF and PSD‐95 in the hippocampus. ^*^ *p* < .05, ^**^ *p* < .01, ^***^ *p* < .001 versus control male; ^$$^ *p* < .01, ^$$$^ *p* < .001 versus control female; ^#^ *p* < .05, ^##^ *p* < .01, ^###^ *p* < .001 versus MSD male; ^&^ *p* < .05, ^&&^ *p* < .01, ^&&&^ *p* < .001 versus MSD female.

The mRNA and protein levels of BDNF and PSD‐95 were significantly different among the three groups (mRNA: BDNF: treatment: *F*_(2,42)_ = 26.91, *p* < .001, sex: *F*_(1,42)_ = 0.51, *p* > .05, treatment × sex: *F*_(2,42)_ = 0.40, *p* > .05; PSD‐95: treatment: *F*_(2,42)_ = 13.01, *p* < .001, sex: *F*_(1,42)_ = 0.01, *p* > .05, treatment × sex: *F*_(2,42)_ = 0.10, *p* > .05; Figure 4a and b; protein: BDNF: treatment: *F*_(2,30)_ = 161.10, *p* < .001, sex: *F*_(1,30)_ = 0.02, *p* > .05, treatment × sex: *F*_(2,30)_ = 0.03, *p* > .05; PSD‐95: treatment: *F*_(2,30)_ = 57.29, *p* < .001, sex: *F*_(1,30)_ = 0.01, *p* > .05, treatment × sex: *F*_(2,30)_ = 0.05, *p* > .05; Figure 4d and e). *Post‐hoc* analysis revealed that EE reversed the MSD‐associated decreased in mRNA and protein levels of BDNF and PSD‐95 in the hippocampus. There were no differences in these results between male and female offspring.

## 4 DISCUSSION

This study confirmed the beneficial effects of EE on MSD‐induced cognitive deficits in CD‐1 mice and its associated mechanisms. The results showed that EE exposure significantly improved the decline of hippocampal learning and memory in MSD‐induced offspring. EE reversed the dysfunction of histone acetylation caused by MSD, as shown by increased expression of CBP, acetyl‐H3K9, acetyl‐H4K12, and decreased expression of HDAC2, in hippocampal tissues. Increased expression levels of BDNF and PSD‐95 indicated that EE had beneficial effects on synaptic plasticity markers. It suggests that a housing environment could be a significant nonpharmacological method for improving cognitive dysfunction through the histone acetylation pathway.

### 4.1 An enriched environment improved the spatial cognition impairment resulting from maternal SD

MSD, as a prenatal stress, affects the development of the fetal central nervous system, especially the hippocampus, through interfering with the intrauterine environment. The Morris water maze test was chosen as a robust and reliable test that is strongly correlated with spatial learning and memory function (Zhuang et al., 2021). The results showed that adult CD‐1 offspring of male and female mice (90‐day‐old) whose mothers exposed to SD by activity wheel for 6 h per day during gestational days (GD) 15–21, spent a significantly longer time locating a hidden platform and less time exploring the target quadrant than the control group. Similarly, our previous study demonstrated that young CD‐1 offspring mice (60‐daysold) received the same treatment performed poorly during the Morris water maze test (Zhang et al., 2022). These results indicated that MSD caused spatial cognitive impairment in offspring that persists from adolescence to adult. Furthermore, male offspring Wistar rats (21‐days‐old) exposed to maternal SD using a modified small‐platform method on GD 18, but not on GD 4 and GD 9, for 72 h took significantly longer to find the hidden platform than the controls during the Morris water maze test (Zhao et al., 2014). These results appear to indicate that only SD during late pregnancy could negatively affect the offspring's cognitive function. However, young adult offspring Sprague–Dawley rats exposed to SD with gentle handling for 6 h per day during the first (GD 1–7), second (GD 8–14), and third trimester (GD 15–21) of pregnancy showed dramatic deficits in spatial learning and memory in the Morris water maze test (Peng et al., 2016). These inconsistent results regarding the effects of SD at different stages of pregnancy on the offspring's cognitive ability may be attributed to different strains, methods, and duration of SD. Notably, the above‐mentioned results provide guidance that, no matter what stage of pregnancy sleep disorders occur in, they are worthy of attention and prevention.

EE could improve impaired synaptic plasticity and cognitive dysfunction by increasing synaptic activity in the hippocampus through the exploration of novel objects and social communication. One study showed that a short‐term exposure to EE alleviated spatial learning and object‐place associative memory impairment in a schizophrenia model induced by MK‐801 (Murueta‐Goyena et al., 2019). Another study suggested that EE paradigm with a duration of 13 days reversed maternal separation‐induced impairments of spatial learning and memory function (Joushi et al., 2021). Consistently, the results showed that access to EE from postnatal day (PND) 21–90 improved MSD‐induced cognitive decline in adult offspring. The results are in accordance with our previous study, which showed that the cognitive dysfunction in young offspring suffered from MSD was improved by EE from PND 21–60 (Zhang et al., 2022). Taken together, EE could be used as an intervention to improve spatial learning and memory impairment in offspring whose dams underwent SD during the third trimester of pregnancy.

### 4.2 Enriched environment reversed histone acetylation dysfunction resulting from maternal SD

Accumulating evidence indicates that histone acetylation regulates the transcription of learning and memory‐associated protein genes, including *Homer*, *Arc*, *c‐fos*, and *Creb*, and abnormal histone acetylation is an important factor in the pathogenesis of cognitive impairment (Rudenko & Tsai, 2014). CBP is one of the most well‐studied histone acetylases and has been shown to be critical for long‐term memory formation. Early research indicates that genetic mutations in CBP are linked to cognitive deficits in Rubinstein–Taybi syndrome (Oike et al., 1999). CBP could broadly affect the transcription of genes involved in neuronal proliferation and differentiation, and synaptic plasticity. During in vitro study, the cultured neurons with CBP ablation resulted in impaired outgrowth, immature spines, and deficient activity‐dependent synaptic remodeling (Del Blanco et al., 2019). During in vivo studies, researchers tried to use different methods to knock out CBP to explore the action of CBP on cognitive function and its specific mechanisms. The *CBP^+/−^* heterozygous mice exhibit a severe defect in long‐term LTP of the hippocampus, paralleling their deficits in long‐term memory during fear conditioning and novel object recognition tasks. Moreover, *CaMKIIα‐cre/CBP^f/f^* mice with the CBP gene deleted in the forebrain have impaired long‐term recognition memory during novel object recognition test (Alarcón et al., 2004; Valor et al., 2011). Furthermore, CBP plays a dual role in CREB‐mediated genes as a scaffolding protein to recruit CREB and as a histone acetylase activity (HAT) that can leave epigenetic marks on the chromatin. The *CBP^KIX/KIX^* mice with mutations in the CREB‐binding (KIX) domain of the coactivator CBP show impaired long‐term memory during contextual and cued fear conditioning (Wood et al., 2006), while *CBP{HAT^−^}* mice carrying a dominant‐negative CBP transgene that specifically blocks HAT show deficits in spatial and recognition memory (Korzus et al., 2004). Even though these transgenic mice produce different performances in various learning and memory tasks owing to different gene knockout techniques, it is undeniable that CBP expression level is closely related to cognitive function. Many studies reported that the downregulation of CBP is associated with cognitive impairment in Alzheimer's disease and Huntington's disease (Ettcheto et al., 2018; Giralt et al., 2012). The results of WB and RT‐PCR showed that MSD decreased CBP expression as well as acetyl‐H3K9 and acetyl‐H4K12.

HDAC2 acts in an opposite role to CBP and inhibits learning and memory‐associated gene transcription by repressing the interaction between transcription factor and chromatin. Previous study demonstrates that overexpression of HDAC2 decreases dendritic spine density, synapse number, synaptic plasticity, and associative learning and memory formation (Guan et al., 2009). Inversely, the knockdown of HDAC2 in the hippocampus of CK‐p25 mice, a mouse model of Alzheimer's disease, leads to a rescue of synaptic plasticity, and abolishes the impairment of neurodegeneration‐associated memory (Gräff et al., 2012). The upregulation of HDAC2 and HDAC3 has been observed in the hippocampus of offspring exposed to sevoflurane during late pregnancy on PND 1 and PND 35 accompanied with long‐term cognitive impairment. The current study demonstrated that mRNA and protein levels of HDAC2 were increased in offspring from the MSD group.

Substantial evidence suggests that histone deacetylase inhibitors, including Trichostatin A and sodium butyrate, have been shown to increase the level of histone acetylation to improve cognitive decline in different pathological models (Hsing et al., 2015; Topuz et al., 2020). Additionally, EE improved traumatic brain injury‐ and sevoflurane‐induced cognitive impairment by increasing CBP expression and decreasing HDAC2 expression, respectively (Wang et al., 2018; Yu et al., 2020). Furthermore, mice with CBP deficiency showed a strong defect in EE‐induced neurogenesis and enhancement of spatial navigation (Lopez‐Atalaya et al., 2011). In the present study, the results showed that EE treatment increased CBP and decreased HDAC2 in the offspring from the MSD + EE group. Taken together, EE treatment could reverse histone acetylation dysfunction induced by MSD.

### 4.3 An enriched environment reversed changes in synaptic plasticity markers resulting from maternal SD

BDNF is an important neuroprotective factor associated with cognition and its transcription is controlled by histone acetylation modification. Three days of SD reduced H3 and H4 acetylation levels in the promoters of *Bdnf* and significantly downregulated BDNF expression (Duan et al., 2016). In this study, the expression levels of acetyl‐H3K9, acetyl‐H4K12, and BDNF were decreased in MSD‐induced offspring. Previous study reported that exercise improves *Bdnf* gene transcription and decreases the binding of HDAC2 to the *Bdnf* promoter in the hippocampus, resulting in an increase in BDNF release (Sleiman et al., 2016). Our recent study showed that EE improved the downregulation of expression levels of BDNF induced by MSD in the hippocampus of young offspring mice. In the current study, EE treatment consistently increased the expression levels of acetyl‐H3K9, acetyl‐H4K12, and BDNF in the hippocampus of adult offspring from the MSD + EE group. Meanwhile, PSD‐95 is an important postsynaptic protein associated with synaptic maturation and is involved in postsynaptic plasticity; MSD decreased the mRNA and protein expression of PSD‐95 in the offspring hippocampus, while EE reversed it. These results are in accordance with previous studies showing that exposure to an EE protocol improves sepsis‐associated cognitive impairment by upregulating the expression of PSD‐95 (Córneo et al., 2022).

Several studies have reported that early‐life stress has a sex‐dependent effect on offspring mice. For example, repeated maternal separation impaired synaptic plasticity and cognitive function in male offspring mice, but not in female offspring mice (Talani et al., 2023). Prenatal restraint stress increased anxiety‐like behavior in offspring male rats and ameliorated anxiety‐like behavior and cognitive impairment in female rats (Zuena et al., 2008). In the present study, there were no sex differences in the outcome of MSD and/or EE on the cognitive function of adult offspring mice and its associated molecular mechanisms, which is consistent with our previous study, which showed that the effects of MSD on the cognitive function and synaptic plasticity‐associated proteins were similar between the male and female young offspring mice (Zhang et al., 2022). However, one study reported that the MSD‐induced alteration in the sexual behavior was significantly different between the male and female offspring rats (Alvarenga et al., 2013). The inconsistent results may be attributed to the time and duration of SD, frequency, conditions of the SD, strain, as well as individual characteristics such as age.

Our study has limitations. First, we did not set an additional Control + EE group because the beneficial effects of EE on cognition and synaptic plasticity have been reported previously. Second, histone acetylation is regulated by histone HATs and HDACs, but we only examined CBP and HDAC2. Third, we did not use the chromatin immunoprecipitation technique to specifically detect histone acetylation levels in the promoter region of *Bdnf* and *PSD‐95*. Finally, we did not use histone deacetylases inhibitors to reverse the cognitive impairment induced by MSD to further verify the origin of the cognitive decline is histone acetylation dysfunction.

## 5 CONCLUSION

The present study demonstrated a mechanism by which exposure to SD for 6 h during the third trimester of the pregnancy caused histone acetylation dysfunction and changes in synaptic plasticity markers. Furthermore, EE treatment improved MSD‐induced cognitive impairment through histone acetylation and synaptic plasticity markers in the offspring. This study provides evidence that histone acetylation could be a potential target for the treatment of cognitive decline caused by MSD.

## AUTHOR CONTRIBUTIONS

All authors contributed to the study conception and design. Yue‐Ming Zhang, Ru‐Meng Wei, and Ming‐Zhu Ni: conceptualization, methodology, investigation, writing original draft, formal analysis; Qi‐Tao Wu and Yun Li: investigation, data curation; Yi‐Jun Ge and Xiao‐Yi Kong: methodology, software, visualization; Xue‐Yan Li: visualization, investigation, writing—review & editing; Gui‐Hai Chen: conceptualization, resources, writing—review & editing, supervision, funding acquisition. All authors read and approved the final manuscript.

## CONFLICT OF INTEREST

The authors report no conflict of interests.

### PEER REVIEW

The peer review history for this article is available at https://publons.com/publon/10.1002/brb3.3018.

## Data Availability

The datasets generated during and/or analyzed during the current study are available from the corresponding author on reasonable request.
